# Psychometric Properties of Parental Burnout Assessment and Prevalence of Parental Burnout: A Person-Centered Approach

**DOI:** 10.1016/j.ijchp.2021.100280

**Published:** 2021-11-20

**Authors:** Natalia Suárez, José Carlos Núñez, Rebeca Cerezo, Pedro Rosário, Celestino Rodríguez

**Affiliations:** a Department of Psychology. University of Oviedo, Spain; b Department of Psychology. University of Minho, Portugal

**Keywords:** Parental burnout, Person-centered approach, Prevalence, Parental Burnout Assessment (PBA), Ex post facto study, Burnout parental, Perspectiva personal, Prevalencia, Parental Burnout Assessment (PBA), Estudio ex post facto

## Abstract

**Background/Objetive:**

The objective of this research is threefold. First, to study the structure of the Parental Burnout Assessment (PBA); second, to learn whether parents combine the dimensions of PBA in profiles; and third, to analyze the prevalence levels of parental burnout.

**Method:**

To address these objectives, the responses of 438 mothers and fathers were analyzed with confirmatory factor analysis and latent profile analysis.

**Results:**

Strong evidence of validity (structural) and reliability (internal consistency) of the PBA was found. Four parental burnout profiles were identified. Moreover, from a variable-centered perspective and a person-centered perspective, very high levels of parental burnout were found.

**Conclusions:**

Data indicate that the PBA is a reliable and valid instrument and suggest that practitioners may use the particular scores of the dimensions or the overall score. Likewise, the level of the four dimensions in the four parental burnout profiles (PBP) is similar within and different between profiles. Finally, the prevalence level of parental burnout is very high (over 26%) compared to data from previous studies (3.2%).

## Abstract (es)

**Antecedentes/Objetivo:**

Con esta investigación se pretendió estudiar la estructura del Parental Burnout Assessment (PBA), averiguar si los padres combinan las dimensiones del PBA en términos de perfiles y, finalmente, analizar los niveles de prevalencia.

**Método:**

Se analizaron las respuestas de 438 madres y padres con análisis factorial confirmatorio y análisis de perfiles latentes.

**Resultados:**

Se obtuvo evidencia tanto de validez (estructural) como de fiabilidad (consistencia interna) del PBA. En base a las cuatro dimensiones del PBA se identificaron cuatro perfiles de agotamiento. Tanto desde una perspectiva centrada en la variable como desde una perspectiva centrada en la persona, se obtuvieron niveles muy altos de burnout parental.

**Conclusiones:**

Los resultados del estudio indican que el PBA es un instrumento válido y fiable (pudiéndose utilizar tanto los puntajes particulares de las dimensiones como el puntaje general). Asimismo, el nivel de las cuatro dimensiones, en los cuatro perfiles de burnout parental, es similar dentro de cada perfil, pero diferente entre los perfiles. Finalmente, en comparación con el porcentaje indicado por estudios previos (3,2%), el nivel de prevalencia de burnout parental derivado de nuestro estudio es muy alto (sobre un 26%).

The COVID-19 outbreak has led to an unprecedented crisis. In Europe, the spread of the virus was quick, and Spain became the second epicenter by number of cases and deceased (Henríquez et al., 2020). People were confined at home since the middle of March for two months. The levels of anxiety, depression and especially stress, increased through the confinement caused by the COVID-19 (Bueno-Novitol et al., 2021; Planchuelo-Gómez et al., 2020). Year 2020 has been a challenging year to children, teenagers, and families due to COVID-19 pandemic. Isolation, contact restrictions and economic shutdown affected children, adolescents, and their families in an exceptional way (Fegert et al., 2020).

Schools were closed too until the end of the school year. As a result, children's school tasks and their changes in humor due to the confinement, have increased parents pressure and difficulties to deal with family routines. Parents all over the world experienced a collision of roles –parent, employee, employer, teacher, etc. (Davis et al., 2020). While everybody was confined, parents may be anxious about their economic future, so studying at home is not easy, especially for children with low learning motivation. All family members had to cope with the stress of confinement and social distancing (Fegert et al., 2020) added to the closure of the economic activity. For example, unemployment rates in Spain have gone up to 15.33% in the second quarter of 2020 from 13.78% in the last quarter of 2019 (Henríquez et al., 2020).

Then, although the confinement situation may include positive factors for parents (e.g., the ability to spend more time with their family and children), several features of the COVID-19 emergency may increase the risk of trauma (Davis et al., 2020), including the loss of predictability in the known world, immobility, detachment or distancing, a lost sense of time, and a lost sense of security (Fontanesi et al., 2020). In a study carried out in the Czech Republic, it was showed that most children spent during the confinement 2-4 hours a day studying, while parents help them at least half the time. Parents mostly explain task instructions, check the work their children have done, and teach new topics. In this same study, parents reflected that their main difficulties regarding the home education situation are the lack of devices, the lack of time and the lack of expertise. During this pandemic, some parents could experience stress, anxiety, fatigue, and many other symptoms that look like as burnout, a kind of burnout not caused by work but by parenting. In a recent study, Davis et al. (2020), based on multiwave panel data from the National Panel Study of Coronavirus pandemic (NPSC-19), find that parents with children who struggled with distance learning experienced elevated mental distress. Given that the coronavirus is still with us, and we do not know to what extent the level of stress for parents may continue to be present, Davis et al. (2020), warn of the importance of supporting parents during this time to improve students' schooling.

Burnout was first defined in the professional setting as “the mental and physical exhaustion of working permanently in touch with others” (Maslach, 1976). Years later, some similarities were found between burnout, professional exhaustion, and the difficulties experienced by adults in their parental roles (Freudenberger, 1974; Maslach & Jackson, 1981). As recent research from Núñez et al. (2021) alerts, parenthood is nowadays one of the most challenging tasks that adults face during their lives.

Parental burnout (PB) has been receiving growing attention in the last few years. In fact, Mikolajczak and Roskam (2020) found that the number and frequency of burnout symptoms are direct indicators of the severity of burnout and are likely to predict its pervasive consequences. Moreover, recent research has shown that the levels of anxiety, depression, and especially stress have significantly increased through the confinement caused by the COVID-19 pandemic (Bueno-Novitol et al., 2021; Planchuelo-Gómez et al., 2020). However, PB may not be confounded with parental stress (Mikolajczak & Roskam, 2020). Parents are likely to feel PB when their stress overwhelms their resources to cope with situations. Besides, individuals’ PB manifests through four main symptoms: exhaustion; overload, and loss of pleasure with parenting; emotional distancing from one's children; and contrast between the parent you are and the parent you wanted to be (Roskam et al., 2018; Roskam et al., 2021). For example, extant research has found that parents who do not enjoy being with their children will have difficulties in parenting (Martínez-Vicente et al., 2020; Pérez-Fuentes et al., 2021; Séjourné, Sánchez-Rodríguez, Leboullenger, & Callahan, 2018).

PB has generally been measured with instruments derived from the Maslach Burnout Inventory (MBI), such as the Parental Burnout Inventory (PBI; Roskam et al., 2017). In 2017, Roskam et al. claimed that a three-dimensional structure was best suited to represent PB, and in 2018 carried out a new study using an inductive method to analyze the construct (Roskam et al., 2018). These authors examined the testimonies of fathers and mothers with high levels of PB. Data from this research indicate a parental exhaustion syndrome including four dimensions: exhaustion in one's parental role, contrast with one's previous parental self, feelings of being fed up with one's parental role, and emotional distancing from one's children. The new instrument with 23 items (Parental Burnout Assessment, PBA) has shown very good reliability scores (Exhaustion in parental role: 9 items, α = .93; Contrast in parental self: 6 items, α = .94; Feelings of being fed up: 5 items, α = .91; Emotional distancing: 3 items, α = .77), as well as factorial validity and invariance between gender and academic subjects.

In the last two years, researchers from different countries and cultures have tried to verify the structure of the PBI. One of the largest studies has been carried out by Roskam et al. (2021), gathering data from 42 countries on five continents (a total of 17,409 fathers and mothers). Findings showed that the PBI has excellent reliability as a general measure of PB (α ≥ .85 in all participating countries), as well as adequate construct validity in all countries. However, researchers found that the cultural factor (greater or lesser individualism) affected the prevalence level of PB (the higher the cultural individualism, the higher the PB level). The prevalence ranged between 0% in Cuba, Peru, Turkey, and Thailand and 8.4% in the USA, with 7.9% in Belgium and 7.3% in Poland. According to this study, the prevalence of PB in Spain is 3.2%.

While an evaluation instrument for job burnout was developed very early, no specific measurement instruments were developed for assessing PB until very recently. To the best of our knowledge, the Parental Burnout Inventory (PBI; Roskam et al., 2017) was the first, but the Parental Burnout Assessment (PBA; Roskam, et al., 2018) is currently the most used by researchers (e.g., Roskam et al., 2021). In the last two years, the PBA has been used in an important number of investigations, both to analyze its psychometric properties in different countries and to examine the relationships between PB and individual and contextual variables (e.g., Aunola et al., 2020; Cheng et al., 2020; Roskam et al., 2021; Szczygiel et al., 2020). For example, Roskam et al. (2021) carried out a macro-study in 42 countries to analyze the validity of the PBA and learn the prevalence of parent PB. Findings showed invariance regarding the structural validity (the structural model of the PBA with four first-order factors and a general second-order factor fits data from all countries), and large differences in the prevalence of the factors found (e.g., prevalence was higher in countries with an individualistic culture).

However, these investigations have been carried out from a variable-centered approach. Following this perspective, PB scores derive from the sum of the four factors’ direct scores. Still, as occurs in other fields of psychology and education (e.g., Pastor et al., 2007), the study of PB from a person-centered approach can provide an ecological and realistic perspective. Individuals’ behaviors are likely to respond to configurations resulting from the combinations of the different dimensions of PB, rather than to singular variables. However, a group of people may combine these variables in the same fashion in a profile. The latter way of approaching measuring the construct may be understood as a person-centered approach. The present research has been carried out from a person-centered statistical approach.

The data gathered in this study are expected to improve the current understanding of the PB construct when analyzed from a person-centered approach. To this aim, the procedures were as follows. First, we ran a confirmatory factor analysis on the validity of the PB construct (as defined by Roskam et al., 2018). Second, a Latent Profile Analysis (LPA) examined the potential profiles of PB based on the four dimensions of the construct. Finally, we analyzed the prevalence of PB, both from a variable-centered and from a person-centered approach.

## Method

### Participants

This study was conducted using snowball sampling, immediately after the 2020 confinement (by Sars-Cov-2). The link to the questionnaire, to be completed online, was sent by email and via WhatsApp. The questionnaire was available to complete during the first weeks of June and took about 10 minutes. All the questions required responses. Participants were 438 Spanish parents who completed the questionnaire about themselves, their family and children. Most participants were mothers (90.2%) and they mostly were between 35 and 44 years old. From the participants, 21.9% had one child from 0 to 3 years old, 41.6% had one child from 4 to 10 years old, and 36.5% had one child from 10 to 16 years old. In most cases (89.2%) both parents lived together at home. Most of the participants have university studies (69.2%) and live in houses of more than 50 m^2^ (98.7%). During confinement, mothers continue to work in the company, or telework, to a lesser extent than fathers (55.3% and 75.6%, respectively). The study met ethical standards of the Helsinki Declaration (Williams, 2008) and has been approved by the Ethics Committee of the University of Oviedo (COPRAMO240/18). Participation in the study was voluntary, and the anonymity and ethical treatment of the data were guaranteed.

### Instruments

Data were collected online through a link. The questionnaire could be completed using a smartphone or any other internet-connected device. It included a section for participants to provide informed consent. The questionnaire included, among other variables, the Parental Burnout Assessment (PBA; Roskam et al., 2018). The PBA has four factors: (i) Exhaustion in one's parental role (9 items; α = .94, ω = .94, *r*_xx_ = .95; e.g., “I feel completely run down by my role as a parent”), (ii) Contrast with previous parental self (6 items; α = .93, ω = .93, *r*_xx_ = .90 ; e.g., “I don't think I'm the good father / mother that I used to be to my child (ren)”), (iii) Feelings of being fed up with one's parental role (5 items; α = .90, ω = .91, *r*_xx_ = .88; e.g., “I can't stand my role as father / mother anymore”), and (iv) Emotional distancing from one's children (3 items; α = .77, ω = .78, *r*_xx_ = .82; e.g., “I do what I'm supposed to do for my child (ren), but nothing more”). Participants responded on a 5-point scale from 1 (*never*) to 5 (*always*). Roskam et al. (2018) obtained evidence of an acceptable fit of this model (CFI = .94; TLI = .93; RMSEA = .07; SRMR = .04).

### Statistical analyses

Data was analyzed in several phases. Using Mplus7.11 (Muthén & Muthén, 1998-2012), confirmatory factor analyzes were run to examine the factorial structure of the Spanish version of the PBA. Two models were fit: unidimensional and multidimensional (i.e., factorial structure of four factors similar to that obtained by Roskam et al., 2018). Afterwards, models fit was assessed as follows: the statistics AIC, BIC, and SSABIC were used to select the best model, and the statistics χ^2^, CFI, TLI, RMSEA, and SRMR were used to analyze the fit model. The best model is the one with lower AIC, BIC, and SSABIC, showing χ^2^ *p* > .05, CFI and TLI ≥ .95, and RMSEA and SRMR ≤ .60. The selection of the best model was carried out based on different criteria as follows: the formal test of the adjusted maximum likelihood ratio of Lo, Mendell, and Rubin -LMRT, the Akaike information criteria (AIC), the Bayesian of Schwarz (BIC), and the BIC adjusted for the sample size (SSA-BIC), as well as the value of the entropy and the size of each subgroup or class. Significant p-values associated with LMRT indicated significant improvement in model fit regarding the solution with one less class. Lower AIC, BIC, and SSA-BIC values indicate a better fit of the model. These criteria should complement the information provided by the formal conditional adjustment test. Likewise, it should be noted that small classes (*n* < 5%), although typically considered spurious classes, sometimes constitute a profile of interest. In order to determine the classification accuracy of the selected model, the posteriori probabilities and the entropy statistic were calculated. This statistic takes values between zero and one; the closer it is to one, the more accurate the classification is (values higher than .80 indicate good classification quality). Finally, for selecting the best model, the theoretical significance of the classes, or profiles, was taken into account. For CFA and LPA, maximum likelihood estimation with robust standard error (MLR) was used.

Finally, our best PBA model was the multifactorial one. Afterwards, based on the four dimensions of PBA, a Latent Profile Analysis (LPA) was carried out to group parents based on their potential profiles of burnout (Lanza et al., 2003). When fitting the models, due to its potential interference on data, gender was included as a covariate (effect of gender on the establishment of classes and the allocation of individuals to them). SAVE = CPROBABILITIES was included in the Mplus syntax to create a variable with the assignment of the subjects to the groups.

## Results

### PBA factorial structure

Table 1 shows the descriptive statistics and Pearson's correlation matrix corresponding to the 23 items of the PBA. All correlations between items were statistically significant at *p* <.001. The mean of the items was between 1.46 and 2.55, and the skewness and kurtosis values were within parameters for normality.

**Table 1:** Pearson correlations and descriptive statistics (mean, standard deviation, asimetría and kurtosis).

	Gender	1	2	3	4	5	6	7	8	9	10	11	12	13	14	15	16	17	18	19	20	21	22	23
Gender	—																							
PBA_1	.16 3⁎⁎	—																						
PBA_2	-.117*	.581	—																					
PBA_3	-.115*	.726	.666	—																				
PBA_4	-.090	.595	.557	.690	—																			
PBA_5	-.143⁎⁎	.442	.629	.591	.643	—																		
PBA-6	-.077	.477	.558	.635	.618	.605	—																	
PBA_7	-.131⁎⁎	.642	.555	.706	.629	.547	.593	—																
PBA_8	-.027	.552	.532	.608	.610	.528	.532	.649	—															
PBA_9	-.054	.564	.589	.698	.663	.587	.765	.648	.614	—														
PBA_10	-.084	.644	.591	.702	.679	.527	.654	.631	.585	.742	—													
PBA_11	.006	.379	.453	.480	.534	.497	.563	.467	.428	.571	.590	—												
PBA_12	-.102*	.598	.590	.693	.671	.582	.631	.785	.622	.698	.686	.563	—											
PBA_13	-.133⁎⁎	.466	.662	.600	.562	.750	.624	.550	.567	.617	.547	.478	.610	—										
PBA_14	.021	.326	.435	.466	.542	.480	.491	.395	.501	.516	.522	.556	.454	.483	—									
PBA_15	-.101*	.575	.596	.684	.649	.588	.585	.702	.642	.643	.632	.524	.719	.593	.569	—								
PBA_16	-.034	.506	.578	.625	.605	.560	.817	.566	.518	.774	.700	.613	.626	.588	.488	.609	—							
PBA_17	-.090	.375	.598	.538	.562	.720	.661	.474	.512	.623	.489	.495	.555	.699	.515	.529	.634	—						
PBA_18	-.097*	.390	.588	.536	.531	.691	.647	.478	.489	.592	.498	.523	.560	.663	.476	.529	.602	.808	—					
PBA_19	-.109*	.448	.669	.600	.593	.706	.638	.524	.578	.634	.567	.525	.582	.741	.556	.588	.608	.796	.751	—				
PBA_20	-.006	.320	.488	.460	.481	.474	.521	.408	.421	.503	.436	.471	.428	.484	.439	.450	.521	.545	.540	.604	—			
PBA_21	-.072	.550	.526	.635	.641	.508	.597	.688	.528	.632	.656	.509	.718	.539	.487	.685	.622	.482	.505	.533	.445	—		
PBA_22	.009	.400	.526	.506	.552	.570	.521	.479	.598	.547	.541	.507	.523	.613	.584	.567	.524	.581	.579	.682	.567	.588	—	
PBA_23	-.036	.540	.512	.597	.601	.515	.519	.631	.981	.606	.578	.425	.608	.564	.495	.633	.504	.496	.470	.564	.401	.518	.579	—
*M*	1.10	2.53	1.85	2.28	2.08	1.87	1.52	2.55	2.30	1.82	1.97	1.61	2.26	1.78	1.80	2.14	1.50	1.54	1.71	1.65	1.46	2.01	2.04	2.30
*SD*	0.29	1.22	1.03	1.14	1.06	1.03	0.85	1.14	1.06	1.04	1.07	0.90	1.05	0.96	0.95	1.06	0.84	0.87	0.99	0.90	0.84	1.01	1.04	1.05
Skewness	2.71	0.20	0.96	0.45	0.60	0.94	1.52	0.21	0.23	1.09	0.95	1.43	0.35	0.95	0.99	0.44	1.75	1.69	1.32	1.23	1.83	0.66	0.68	0.17
Kurtosis	5.37	-0.96	0.14	-0.55	-0.40	-0.01	1.62	0.68	-0.77	0.50	0.32	1.57	-0.52	0.02	0.32	-0.76	2.87	2.59	1.06	0.69	2.86	-0.22	-0.22	-0.91

*Note*: PBA_1 to PBA_23 are the ítems of Parental Burnout Assessment (PBA), and their intercorrelations are statistically significant at *p* < .001. In the correlations between gender and PBA ítems:

⁎ = *p* < .05; ^⁎⁎^*p* < .01. Gender: mother (1) = 90.2%; father (2) = 9.8%.

Three factorial models were fit: one-dimensional –PBA_1F- (a single factor explains the variability in the 23 items), multidimensional –PBA_4F- with four first-order factors (Exhaustion -9 items-, Contrast in parental self -6 items-, Feelings of being fed up -5 items-, and Emotional distancing -3 items), and hierarchical –PBA_J- with four first-order factors and one second-order factor (general PBA). Data show an evident lack of fit of the three models: unidimensional (χ^2^_230_ = 2826.01, *p* < .001; CFI = .736; TLI = .710; RMSEA = .161; SRMR = .066), multidimensional (χ^2^_224_ = 2030.18, *p* < .001; CFI = .816; TLI = .792; RMSEA = .136; SRMR = .050), and hierarchical (χ^2^_226_ = 2075.62, *p* < .001; CFI = .812; TLI = .789; RMSEA = .137; SRMR = .055). However, considering the values of AIC and BIC, the first order multidimensional model (PBA_4F) was the best fit model of the three: PBA_1F (AIC = 2911.55; BIC = 3099.33), PBA_4F (AIC = 2134.18, BIC = 2346.46), PBA_J (AIC = 2175.62, BIC = 2379.73).

Acknowledging the information provided by the modification indices and the residuals, we learned that the model's lack of a good fit was mainly due to the existence of covariance between some variances. Therefore, the best fitting model (PBA_4F) was re-estimated after including some covariances. The result shows a good fit of the model (χ^2^_207_ = 501.93, *p* < .001; CFI = .970; TLI = .963; RMSEA = .057; SRMR = .036). Consequently, the results suggest the acceptance of the multidimensional model as adequate.

Table 2 shows that the variability of each of the 23 items is significantly explained by their respective theoretical factors. It is observed that the factorial weights are high in most of the items. On the other hand, the reliability of the subscales is high for exhaustion in parental role, contrast in parental self, and feelings of being fed up; and acceptable for the emotional distancing subscale.

**Table 2:** Factor loadins estimates in CFA from the four-factor solution of Parental Burnout Assessment (PBA) (*N* = 483).

						95% Confidence Interval	
Factor	Indicator	Estimate	SEa	*Z*-value	*p*	Lower	Upper
Exhaustion in Parental Role (α = .94) (ω = .94) (*r*_xx_ = .95)	PBA_1	0.836	0.046	18.321	< .001	0.747	0.926
	PBA_4	0.852	0.040	21.040	< .001	0.772	0.931
	PBA_7	0.935	0.041	22.736	< .001	0.854	1.015
	PBA_8	0.783	0.040	19.815	< .001	0.705	0.860
	PBA_10	0.871	0.047	18.455	< .001	0.779	0.964
	PBA_12	0.896	0.039	23.111	< .001	0.820	0.972
	PBA_15	0.866	0.037	23.138	< .001	0.793	0.939
	PBA_21	0.812	0.041	19.577	< .001	0.730	0.893
	PBA_23	0.761	0.037	20.729	< .001	0.689	0.833
Contrast in Parental Self (α = .93) (ω = .93) (*r*_xx_ = .90)	PBA_2	0.789	0.044	18.026	< .001	0.703	0.874
	PBA_5	0.827	0.044	18.967	< .001	0.742	0.913
	PBA_13	0.792	0.046	17.127	< .001	0.702	0.883
	PBA_17	0.749	0.049	15.253	< .001	0.653	0.845
	PBA_18	0.815	0.048	17.014	< .001	0.721	0.909
	PBA_19	0.813	0.039	20.606	< .001	0.736	0.891
Feelings of being fed up (α = .90) (ω = .91) (*r*_xx_ = .88)	PBA_3	0.925	0.042	21.973	< .001	0.842	0.999
	PBA_6	0.669	0.045	14.712	< .001	0.580	0.758
	PBA_9	0.866	0.047	18.422	< .001	0.774	0.958
	PBA_11	0.591	0.044	13.422	< .001	0.505	0.678
	PBA_16	0.652	0.051	12.885	< .001	0.553	0.751
Emotional Distancing (α = .77) (ω = .78) (*r*_xx_ = .82)	PBA_14	0.636	0.048	13.212	< .001	0.542	0.678
	PBA_20	0.576	0.048	12.102	< .001	0.483	0.688
	PBA_22	0.848	0.044	19.358	< .001	0.762	0.811

*Note*: α (Cronbach coeficiente), ω (McDonald coeficient), *r*_xx_ (Spearman-Brown coeficient).

### Latent profile analysis

Table 3 shows the descriptive statistics and the Pearson correlation matrix corresponding to the four dimensions of PBA and gender. In relation to the four dimensions of the PBA, the skewness and kurtosis data indicate a normal distribution. Taking into account the entire sample, the mean data show medium-low levels in the four dimensions of burnout. The correlation matrix shows that mothers, compared to fathers, present a higher level in two of the four factors of the PBA (exhaustion and contrast in parental self). Moreover, the four factors of the PBA were strongly related.

**Table 3:** Pearson correlation matrix and descriptive statistics.

	1	2	3	4	5
1. Gender (1 = mother; 2 = father)	−				
2. Exhaustion in parental role	-.111*	−			
3. Contrast in parental self	-.134*	.748⁎⁎	−		
4. Feelings of being fed up	-.068	.855⁎⁎	.793⁎⁎	−	
5. Emotional distancing	.011	.700⁎⁎	.746⁎⁎	.717⁎⁎	−
*M*	1.09	2.23	1.73	1.74	1.76
*SD*	0.29	0.89	0.83	0.81	0.78
Skewness	2.71	0.48	1.19	1.22	1.00
Kurtosis	5.37	-0.26	0.84	1.12	0.60

⁎ *p* < .05;

⁎⁎ *p* < .01.

Several latent profile models were fitted. The variables for class formation were the four dimensions of the PBA. Two, three, four, and five class models were successively fitted. The process was stopped at the five-class model because it was no better than the four-class model (see Table 4).

**Table 4:** Results of fitting two, three, four, and five latent class model.

	Two-class model	Three-class model	Four-class model	Five class model
AIC	3929.61	3640.12	3421.23	3333.40
BIC	3986.76	3721.76	3527.36	3464.03
SSA-BIC	3942.33	3658.29	3444.85	3362.48
LMRT-test	1037.91⁎⁎⁎	293.45*	224.73*	97.16
Entrophy	.946	.892	.914	.927
n < 5%	0	0	0	1

*Note*: AIC = Akaike Information Criterion; BIC = Schwarz Bayesian Information Criterion; SSA-BIC = BIC adjusted for the sample size; LMRT-Test = adjusted Lo-Mendell-Rubin maximum likelihood ratio test; n < 5% = number of class with less of 5% of individuals.

⁎ *p* < .05; ** *p* < .01;

⁎⁎⁎ *p* < .001.

The four-class model was selected as it presents better fit statistics than the three or the five-class models. On the one hand, the AIC, BIC, and SSA-BIC statistics of the four-class model are lower than those of the three-class model. Furthermore, the LMRT-test of the four-class model was statistically significant, suggesting that this model fits data better than did the three-class model. Likewise, none of the four classes have a number of individuals less than 5%, and the quality of the classification (entropy) of the subjects of the four-class model is better than that of the three-class model. In this model, the parents were very well assigned to the corresponding class (class 1 = .965; class 2 = .934; class 3 = .935; class 4 = .967). On the other hand, although the AIC, BIC, and SSA-BIC statistics of the five-class model were lower than those of the four-class model, the LMRT test indicates that, statistically, the five-class model was no better than the four-class model. However, one of the five classes contains an excessively small number of individuals (1.5%); consequently, the four-class model has been selected as the best fit model.

**Figure 1:**
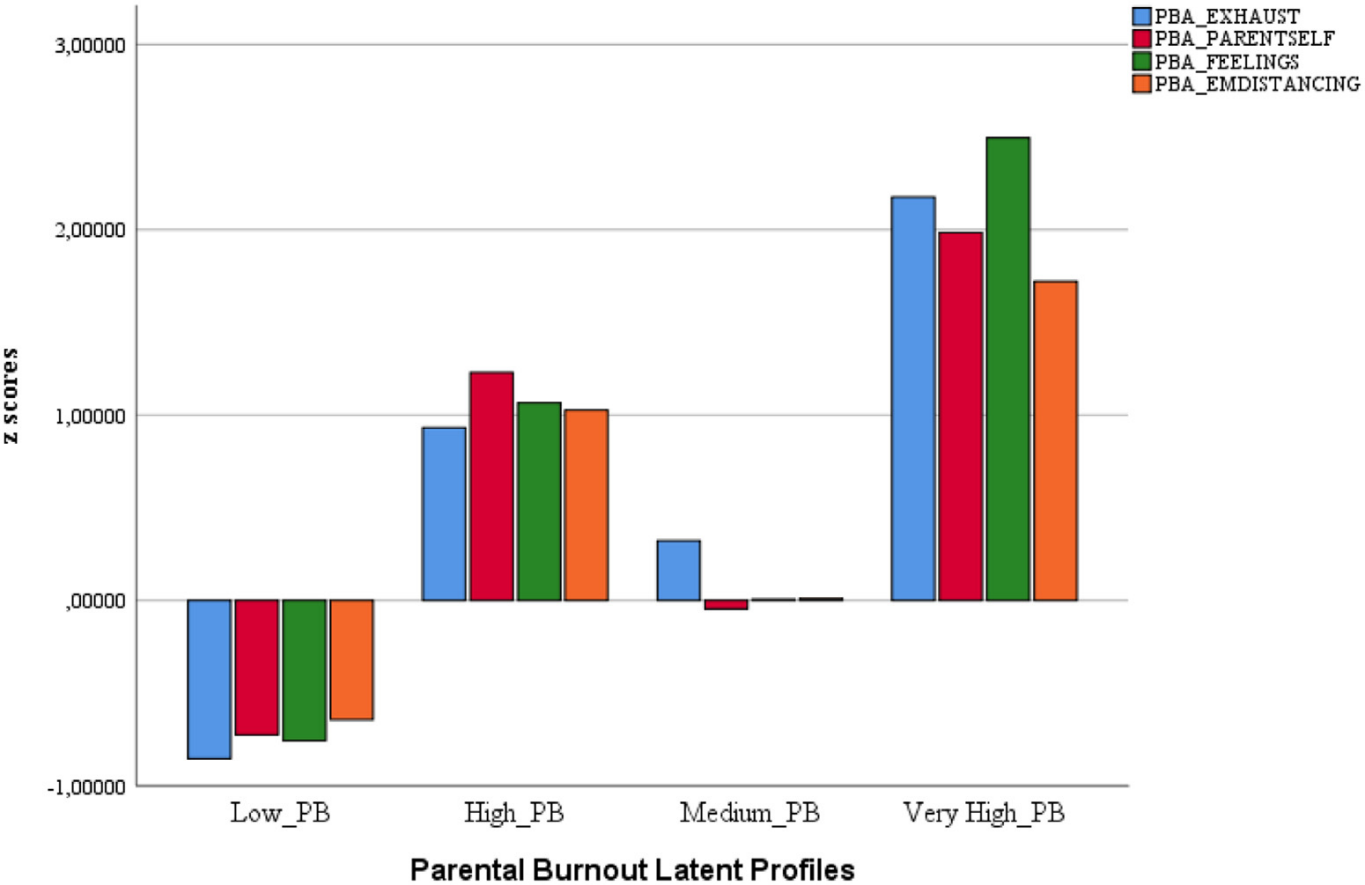
Parental burnout profiles based on the combination of the four dimensions of the Parental Burnout Assessment (PBA): EXHAUST (Exhaustion in one's parental role), PARENTSELF (Contrast with previous parental self), FEELINGS (Feelings of being fed up with one's parental role), and EMDISTANCING (Emotional distancing from one's children).

Table 5 provides the statistics corresponding to the four-class model (profiles), both raw and standardized scores. Figure 1 offers the graphic representation of the four profiles based on the standardized scores.

**Table 5:** Estimates for the PBA dimensions in the model of four clases.

	*RS* (*SD*)	*SS*	LO 5%	HI 5%
Class 1: *Low Parental Burnout Profile (LPBP)*				
Exhaustion in one's parental role	1.48 (0.39)	-0.855	-0.927	-0.783
Contrast with previous parental self	1.13 (0.23)	-0.714	-0.754	-0.674
Feelings of being fed up	1.14 (0.20)	-0.751	-0.784	-0.718
Emotional distancing	1.26 (0.38)	-0.644	-0.705	-0.584
Class 2: *High Parental Burnout Profile (HPBP)*				
Exhaustion in one's parental role	3.07 (0.32)	0.928	0.822	1.033
Contrast with previous parental self	2.76 (0.50)	1.230	1.066	1.394
Feelings of being fed up	2.61 (0.41)	1.059	0.884	1.234
Emotional distancing	2.57 (0.61)	1.025	0.838	1.212
Class 3: *Medium Parental Burnout Profile (MPBP)*				
Exhaustion in one's parental role	2.52 (0.39)	0.324	0.233	0.416
Contrast with previous parental self	1.70 (0.42)	-0.062	-0.171	0.047
Feelings of being fed up	1.75 (0.33)	0.007	-0.091	0.105
Emotional distancing	1.77 (0.56)	0.014	-0.137	0.164
Class 4: *Very High Parental Burnout Profile (VHPBP)*				
Exhaustion in one's parental role	4.18 (0.43)	2.133	1.809	2.457
Contrast with previous parental self	3.39 (0.83)	1.999	1.639	2.358
Feelings of being fed up	3.77 (0.50)	2.460	2.095	2.826
Emotional distancing	3.12 (0.83)	1.729	1.404	1.729

*Note: RS* (Raw Scores: 1 min.,…, 5 max.) and *SD* (standard deviation); *SS* (Standardized Scores: *M* = 0, *SD* = 1).

The four classes correspond to four different profiles, with a very different number of subjects in each (χ^2^ (3, 438) = 153.84; *p* < .001). Specifically, almost half of the sample (46.6%) shows a low profile in the four dimensions of PB (close to a typical score, below the average). This profile was termed Low Parental Burnout Profile -LPBP. Another group of parents was identified (over 28.5%) with a medium PB profile (the standardized scores of the four dimensions of the PBA are around zero). This profile was termed *Medium Parental Burnout Profile* –MPBP. As for the remaining 25% of fathers and mothers that made up the sample of this study, they constitute two different profiles, both sharing levels higher than the expected average with respect to the dimensions of the PBA. Specifically, 18.9% of fathers and mothers were identified as having high levels of burnout (around a standard deviation above the mean), which we termed High Parental Burnout Profile –HPBP. Finally, the remaining 6% of parents show a very high profile in burnout (about two standard deviations above the mean) and, consequently, were termed Very High Parental Burnout Profile –VHPBP. Also note that the four profiles, in the four dimensions, are statistically different: exhaustion (*F* (3, 434) = 644.1; *p* < .001; ηp2 = .817), parental self (*F* (3, 434) = 477.26; *p* < . 001; ηp2 = .767), feelings of being fed up (*F* (3, 434) = 838.55; *p* < .001; ηp2 = .853), and emotional distancing (*F* (3, 434) = 191.02; *p* < .001; ηp2 = .569). Finally, all the comparisons (Bonferroni method) between profiles within each of the four burnout dimensions were statistically significant at *p* < .001.

### Prevalence of PB

The prevalence of PB was estimated based on two procedures, depending on whether a variable-centered perspective (PB) or a person-centered perspective (parental burnout profile) was used. From a variable-centered perspective (PBA total score), Roskam et al. (2021) used the cutoff score of 92 (on a scale from 0 to 138). In our case, since the scale ranges from 23 to 115, the cutoff point was 76. According to this criterion, the prevalence of PB reaches 26.1% (*M* = 84.87; *SD* = 9.42). The current study followed a person-centered perspective; data indicate that around 18.9% of the sampled parents present high and 6% very high levels of PB.

## Discussion

Current data are consistent with those initially provided by Roskam et al. (2018) and those gathered in different countries using the PBA (Roskam et al., 2021). Those findings suggest that PB is a multidimensional construct (Sánchez-Rodríguez et al., 2019). In our study, although both the hierarchical model and the first-order factorial model fit the data well, unlike Roskam et al. (2021) results, we found that the first-order factorial model is the most parsimonious as well as the one with the best fit. Moreover, the reliability data of the four dimensions are consistent with those provided by previous research (e.g., Roskam et al., 2018). Eventually, we may conclude that the four subscales of the PBA are reliable (even emotional distancing, which only has three items).

Evidence was gathered on the existence of groups of individuals with homogeneous PB profiles. Specifically, four intragroup and different between-group homogeneous profiles were found. The most numerous profile, including almost 50% of the sample, includes fathers and mothers showing a low level in the four dimensions of PB. In addition, another important group (almost 30% of the sample) presents average levels of PB. These fathers and mothers, despite the harsh conditions of confinement, have not developed PB. Furthermore, a high number of fathers and mothers show high (18.9%) or very high (6%) levels of PB. Both groups are at risk, but the latter, with 6% of participants showing very high levels of PB, is extremely vulnerable. These prevalence data derived from class analysis is consistent with those gathered from a variable-approach perspective: in both cases, the percentage of fathers and mothers with PB is over 25%. However, this percentage is much higher than the 3.2% reported by Roskam et al. (2021). As the literature suggests (e.g., Bastiaansen et al., 2021; Griffith, 2020), this may be explained by the extreme peculiar conditions experienced between March and May 2020, due to home confinement restrictions imposed by the pandemic COVID-19 (e.g., Bueno-Novitol et al., 2021). However, it is also possible that this negative effect of confinement on PB is more or less high depending on the cultural context in which the data was collected. For example, Mousavi (2020), when investigating the behaviors of Iranian parents, found no effects of the quarantine on PB. Although future post-pandemic studies may provide new information on this matter, these discrepancies may also be due to the methodology used (LPA) for the identification of PB profiles. Or even to the interaction of both factors (confinement conditions and LPA methodology). Future research in this field should help resolve these important discrepancies.

In sum, current data indicate that: (a) the PBA is a valid and reliable instrument; (b) the data derived from the analysis of the factorial structure support both the use of a global score of PB (hierarchical factorial model) and the particular estimation of the scores of the four PB dimensions (first-order factorial model); (c) the four PB profiles (PBP) identified differ notably concerning their level of burnout; (d) approximately 20% of parents have developed high levels of burnout during the 2020 confinement, (e) and finally, 6% of these parents show extremely high levels of PB and are in need of urgent professional help. These parents would benefit from training on emotional competencies to help them improve their well-being (Lin et al., 2021).

Finally, current results should be taken cautiously because our sample is mostly comprised of women (90.2%) and as reported in previous research, the conditions that trigger PB can affect mothers and fathers differently (Bastiaansen et al., 2021; Mousavi, 2020; Roskam et al., 2018). Furthermore, the sample has not been randomly selected, which may affect the degree to which the results can be applied to a larger population. Snowball sampling is a type of non-probability sampling that is used when potential participants are difficult to find or if the sample is limited to a very small subset of the population. Although it is a very good technique for conducting exploratory research, it is not without its limitations. For example, it is generally difficult to determine the sampling error or limits the inferences that can be made about the population to which the sample may belong. For this reason, it is recommended to take the results derived from the present investigation with some caution.
